# Cancer Patient Acceptance of HIV Screening at a Large Tertiary Cancer Center

**DOI:** 10.1093/jncics/pkac055

**Published:** 2022-08-09

**Authors:** Bruno P Granwehr, Kelly W Merriman, Elizabeth Y Chiao, Richard M Grimes

**Affiliations:** Department of Infectious Diseases, Division of Internal Medicine, The University of Texas MD Anderson Cancer Center, Houston, TX, USA; Department of Tumor Registry, Division of Chief Quality Officer, The University of Texas MD Anderson Cancer Center, Houston, TX, USA; Department of Epidemiology, The University of Texas MD Anderson Cancer Center, Houston, TX, USA; Department of General Oncology, The University of Texas MD Anderson Cancer Center, Houston, TX, USA; Division of General Internal Medicine, Department of Internal Medicine, The University of Texas Health Science Center at Houston, McGovern Medical School, Houston, TX, USA

## Abstract

The US Centers for Disease Control and Prevention, the US Preventive Services Task Force, and the National Comprehensive Cancer Network recommend offering HIV testing for patients presenting for cancer care. Not recognizing and treating HIV infection adversely affects both cancer treatment and HIV outcomes. Acceptance rates of oncology patients for HIV screening are not known. Our tertiary cancer center inserted language requesting permission to screen for HIV infection into the consent forms for initial presentation for cancer care. Willingness to undergo testing was examined in 29 549 consecutive new patients. These were analyzed by gender and age. Overall, 80.9% of patients agreed to HIV screening. Incorporation of language requesting permission for HIV screening into the consent form provided at presentation for cancer care relieves clinicians from adding this task.

The United States Preventive Services Task Force (1), the US Centers for Disease Control and Prevention (2), and the National Comprehensive Cancer Network (3) have all recommended that patients should be offered testing for HIV when entering care. This is a particularly important assay for persons with cancer because of the well-established link between HIV infection and AIDS-defining cancers and for other cancers that are more likely to occur in cancer patients (4,5). In addition, it has been demonstrated that cancer patients with HIV infection have much better cancer survival rates if their HIV is treated before they experience significant decline in their immune system (6,7). Obtaining permission to test for HIV can be burdensome on clinicians, who must educate patients and enter into potentially awkward conversations with patients and document test acceptance or refusal. A simple method for removing the burden on clinicians is to request permission for HIV testing at admission to care. Our cancer center adopted this approach for all new patients. This brief communication reports on the willingness of cancer patients to be tested for HIV.

All new patients who entered care between June 11, 2015, and March 3, 2016, were given a form that allows patients to give permission to be screened for HIV (see Figure 1), approved by the Quality Improvement Assessment Board. Forms in which a box was not selected or that were completed manually were excluded from the analysis. The forms were analyzed to determine rates of acceptance of HIV testing and were analyzed by gender and age. Comparisons between these groups, in acceptance of HIV screening, were made using the χ^2^ test (2-sided).

**Figure 1.**
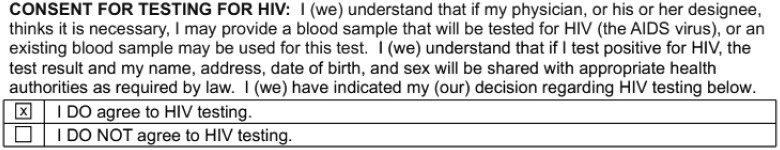
Permission to screen for HIV in consent to diagnosis and treatment. This shows the language included in the institutional consent for diagnosis and treatment, with a check box included, requesting permission for HIV screening. If the patient does not check the box or declines HIV screening, HIV screening may still be completed but includes separate documentation.

Of the 29 549 patients admitted to care during those dates, 80.9% consented to HIV testing. Willingness to be screened for HIV was 71.7% for patients younger than 20 years (which included pediatric patients) and 87.6% among patients aged 20-29 years (see Table 1). For patients older than 18 years, the acceptance rate was 81.0%, but only 70.5% for those younger than 18 years. When comparing all age groups combined, a statistically significant difference in rates of acceptance was shown (*P* < .001). This statistically significant difference remained when directly comparing most age group categories, including all comparisons with patients younger than 20 years (*P* < .001). Of potential interest, however, is that when the youngest 3 age categories (<20 years, 20-29 years, 30-39 years) were removed, a statistically significant difference no longer remained among the remaining age groups, all older than 40 years (*P* = .21). In addition, there was no statistically significant difference in acceptance of HIV screening by gender, with 80.9% of females and 80.8% of males agreeing to HIV testing (*P* = .73; see Table 1).

**Table 1.** Rates of patient agreement with HIV screening, by age and gender

Characteristic	Total No.	No. of patients who agreed (%)	No. of patients who did not agree (%)	*P* ^a^
Age, y				
<20	644	462 (71.74)	182 (28.26)	
20-29	1200	1051 (87.58)	149 (12.42)	
30-39	2452	2076 (84.67)	376 (15.33)	
40-49	4280	3429 (80.12)	851 (19.89)	
50-59	6620	5281 (79.77)	1339 (20.23)	
60-69	7921	6426 (81.13)	1495 (18.87)	
>70	6432	5167 (80.33)	1265 (19.67)	
Total	29 549	23 892 (80.86)	5657 (19.14)	<.001
Gender				
Female	16 509	13 360 (80.93)	3149 (19.07)	
Male	13 040	10 532 (80.77)	2508 (19.23)	
Total	29 549	23 892 (80.86)	5657 (19.14)	.73

a χ^2^ statistic (2-sided test).

Barriers that make physicians reluctant to order HIV testing include lack of patient acceptance, insufficient time for discussion, burdensome consent process, lack of knowledge or training, pretest counseling requirements, competing priorities, and inadequate reimbursement (8). This study demonstrated that there is a high level of acceptance of HIV testing by cancer patients. By having an automatic consent process at admission to care, the physician is relieved of multiple barriers to HIV routine screening. Now that HIV testing is the standard of care as recommended by the United States Preventive Services Task Force, US Centers for Disease Control and Prevention, and the National Comprehensive Cancer Network, the reimbursement issue has also been obviated because HIV testing is the standard of care. An additional incentive for oncologists is that diagnosis of HIV infection in their patients can prevent transmission of this oncogenic virus to future sexual partners as well as current partners, whom patients can encourage to get tested. This may avoid the legal complications that can arise when failure to follow the standard of care results in transmission to another person (9).

As stated by Chiao et al. (4) a dozen years ago, it is “time for oncologists to opt in for routine opt out testing.” In our institution, HIV screening was limited to only 18.6% of cancer patients who were receiving chemotherapy (10). This included 88.4% of non-Hodgkin lymphoma patients, 14% of anal cancer patients, and 9.4% of cervical cancer patients (10). HIV seropositivity was 1.2%, with 0.3% newly diagnosed (10). Without the leadership of oncologists, their patients and the partners of their patients are at risk of worse than anticipated outcomes, potential for second and recurrent cancers, and transmission of an oncogenic virus to others. “Treatment as prevention” has become a mantra in the HIV community, but this may be modified to “treatment as cancer prevention.” As an oncologist would screen renal or liver function prior to initiation of various cancer therapies, screening for HIV should also be considered standard of care. Oncologists should lead the demand for HIV testing at their institutions.

## Funding

No funding was used for this study.

## Notes

**Role of the funder**: Not applicable.

**Disclosures:** The authors have no disclosures.

**Author contributions:** Conceptualization: BG, KM, RG. Methodology: BG, KM, EC, RG. Writing - Original Draft: BG, KM, RG. Writing - Review & Editing: BG, KM, EC, RG.

## Data Availability

Deidentified data may be provided on request to the corresponding author.
